# Drug Interactions With the Ca^2+^-ATPase From Sarco(Endo)Plasmic Reticulum (SERCA)

**DOI:** 10.3389/fmolb.2018.00036

**Published:** 2018-04-11

**Authors:** Francesco Tadini-Buoninsegni, Serena Smeazzetto, Roberta Gualdani, Maria Rosa Moncelli

**Affiliations:** ^1^Department of Chemistry “Ugo Schiff,” University of Florence, Florence, Italy; ^2^Laboratory of Cell Physiology, Institute of Neuroscience, Université Catholique de Louvain, Louvain-la-Neuve, Belgium

**Keywords:** anticancer drug, antimalarial agent, drug-protein interaction, sarco(endo)plasmic reticulum Ca^2+^-ATPase, SERCA activator, SERCA inhibitor, solid supported membrane

## Abstract

The sarco(endo)plasmic reticulum Ca^2+^-ATPase (SERCA) is an intracellular membrane transporter that utilizes the free energy provided by ATP hydrolysis for active transport of Ca^2+^ ions from the cytoplasm to the lumen of sarco(endo)plasmic reticulum. SERCA plays a fundamental role for cell calcium homeostasis and signaling in muscle cells and also in cells of other tissues. Because of its prominent role in many physiological processes, SERCA dysfunction is associated to diseases displaying various degrees of severity. SERCA transport activity can be inhibited by a variety of compounds with different chemical structures. Specific SERCA inhibitors were identified which have been instrumental in studies of the SERCA catalytic and transport mechanism. It has been proposed that SERCA inhibition may represent a novel therapeutic strategy to cure certain diseases by targeting SERCA activity in pathogens, parasites and cancer cells. Recently, novel small molecules have been developed that are able to stimulate SERCA activity. Such SERCA activators may also offer an innovative and promising therapeutic approach to treat diseases, such as heart failure, diabetes and metabolic disorders. In the present review the effects of pharmacologically relevant compounds on SERCA transport activity are presented. In particular, we will discuss the interaction of SERCA with specific inhibitors and activators that are potential therapeutic agents for different diseases.

## Introduction

P-type ATPases are membrane transporters that couple the energy provided by ATP hydrolysis to the active transport of various ions or phospholipids. These enzymes generate and maintain crucial electrochemical potential gradients across biological membranes (Kühlbrandt, 2004; Bublitz et al., 2011). During their enzymatic cycle P-type ATPases form a phosphorylated intermediate by interaction of ATP with a conserved aspartate residue at the catalytic domain.

The Ca^2+^-ATPase from sarco(endo)plasmic reticulum (SERCA), belonging to the P_IIA_-ATPase subfamily, is an intracellular membrane-associated protein of approximately 110 KDa which is involved in cell calcium signaling and homeostasis (Brini and Carafoli, 2009). In muscle cells this enzyme hydrolyzes one ATP molecule to transport two Ca^2+^ ions against their electrochemical potential gradient from the cytoplasm to the lumen of sarcoplasmic reticulum (SR) (Inesi and Tadini-Buoninsegni, 2014). SERCA therefore induces muscle relaxation by pumping back cytosolic calcium into the SR lumen. SERCA isoforms are involved in calcium signaling mechanisms for many biological functions, e.g., excitation-contraction coupling, excitation-secretion coupling, gene transcription, and apoptotic mechanisms. Because of its pivotal role, alterations in SERCA expression and impaired pump function have been related to several diseases, such as Brody's disease, Darier's disease, heart failure, cancer, and diabetes (Brini and Carafoli, 2009).

**Figure 1:**
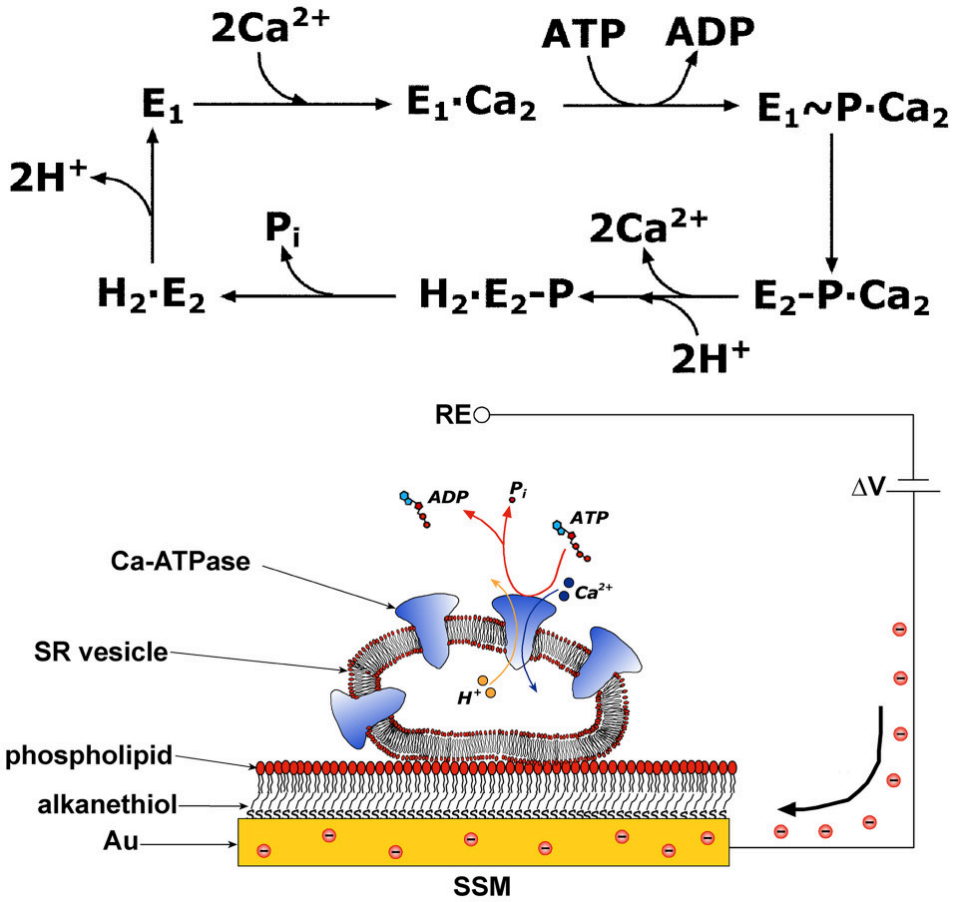
**Upper Panel:** Schematic diagram of sequential reactions in the transport cycle of SERCA. **Lower Panel**: SR vesicle adsorbed on a SSM and subjected to an ATP concentration jump (not drawn to scale). If the ATP jump induces net charge displacement, a compensating current flows along the external circuit (the red spheres represent electrons) to keep constant the potential difference Δ*V* applied across the whole system. RE is the reference electrode. Reprinted from Tadini-Buoninsegni et al. (2008a) with permission from Elsevier.

The SERCA enzyme is one of the best investigated membrane transporter. Its structure comprises three distinct cytoplasmic domains, i.e., the A (actuator), N (nucleotide binding), and P (phosphorylation) domains, and a transmembrane region of 10 helical segments (TM1–TM10) including the two Ca^2+^ binding sites. SERCA transport cycle is described by the E_1_-E_2_ scheme (de Meis and Vianna, 1979). If one starts at the E_1_ state, the ATPase cycle (Figure 1) begins with high affinity binding of two Ca^2+^ ions derived from the cytosol, followed by phosphorylation of the enzyme by ATP and formation of a high energy E_1_~P state. During relaxation from the E_1_~P state to the lower energy E_2_P state, Ca^2+^ ions are translocated across the membrane and released into the SR in exchange for luminal protons. Hydrolytic cleavage of the phosphoenzyme (dephosphorylation) is the final reaction step, which allows the enzyme to undergo a new transport cycle. High resolution crystal structures of various conformational states in the SERCA transport cycle were obtained, as described in detailed reviews (Toyoshima, 2008; Møller et al., 2010; Bublitz et al., 2013; Toyoshima and Cornelius, 2013).

In this short review we will focus our attention on the interaction of SERCA with specific inhibitors and activators that may represent potential therapeutic agents for different diseases. To investigate the effects of pharmacologically relevant compounds on SERCA transport activity, we employ an electrophysiological technique, which is discussed in the next section.

## Drug interactions investigated by electrophysiology based on solid supported membranes

The ion transport mechanism of P-type ATPases, e.g., Na^+^,K^+^-ATPase, SERCA, and Cu^+^-ATPases (ATP7A and ATP7B) (Pintschovius et al., 1999; Tadini-Buoninsegni et al., 2008a; Lewis et al., 2012; Inesi et al., 2014; Tadini-Buoninsegni and Smeazzetto, 2017), was investigated by an electrophysiological technique based on a solid supported membrane (SSM). In particular, SSM-based electrophysiology was useful to identify electrogenic steps and to assign rate constants to partial reactions in the transport cycle of P-type ATPases. In the case of Na^+^,K^+^-ATPase SSM-based electrophysiology provided a direct proof for the electrogenicity of Na^+^ binding to the cytoplasmic side of the protein (Pintschovius et al., 1999). Also in the case of SERCA SSM-based electrophysiology was employed for a detailed characterization of the enzyme's transport cycle, especially as concerns Ca^2+^ binding and Ca^2+^/H^+^ exchange (Tadini-Buoninsegni et al., 2006; Liu et al., 2009).

This technique makes use of a hybrid alkanethiol/phospholipid bilayer supported by a gold electrode (SSM, Figure 1; Pintschovius and Fendler, 1999). The SSM is formed in two sequential self-assembly steps. First, an octadecanethiol monolayer is obtained which is covalently bound to the gold electrode via the sulfur atom. Then, a second phosphatidylcholine monolayer is formed on top of the thiol layer. Proteoliposomes, membrane fragments, or vesicles containing the ATPase are adsorbed on the SSM surface (Figure 1). Once adsorbed, the ATPase molecules are activated by a concentration jump of a specific substrate through fast solution exchange. By rapidly changing from a solution containing no substrate for the protein to one that contains a substrate, the protein is activated and a current transient is detected, which is related to charge displacement across the ATPase. The transient nature of the current signal is a consequence of the capacitively coupled system formed by the SSM and the membrane entities adsorbed on it (Schulz et al., 2008; Tadini-Buoninsegni and Bartolommei, 2016). In the case of SERCA, an ATP concentration jump on SERCA-containing vesicles adsorbed on the SSM generates a current signal, that is related to an electrogenic event corresponding to translocation and release of bound Ca^2+^ upon phosphorylation by ATP within the first enzyme cycle (Tadini-Buoninsegni et al., 2006). Therefore, the SSM technique allows pre-steady state measurements of charge displacements within the first transport cycle of the ATPase, while steady-state currents are not measured.

SSM-based electrophysiology was successfully employed to investigate drug interactions with P-type ATPases. In this respect, the effects of various compounds of pharmacological interest on SERCA pumping activity were characterized by SSM-based current measurements (Tadini-Buoninsegni et al., 2008b, 2017; Bartolommei et al., 2011; Ferrandi et al., 2013; Sadafi et al., 2014). A molecular mechanism was proposed to explain the effect of each compound, and the reaction step and/or intermediate of the pump cycle affected by the drug was identified.

We point out that the SSM electrode combined with robotized instrumentation is an attractive tool for drug screening and development (Kelety et al., 2006). In this respect, high-throughput devices capable of performing automated measurements have been developed. For example, the SURFE^2^R 96SE device (Nanion Technologies, Munich, Germany) is able to analyze 96 SSM sensors in a fully parallel mode allowing determination of the dose dependence of 100 compounds in <30 min (Bazzone et al., 2017).

## Pharmacological inhibitors of SERCA activity

Various SERCA inhibitors with a variety of chemical structures are known (Michelangeli and East, 2011). These compounds represent a very useful tool in studies of the SERCA catalytic and transport mechanism. Using X-ray crystallography of SERCA-inhibitor complexes (Toyoshima and Nomura, 2002; Olesen et al., 2004; Obara et al., 2005; Moncoq et al., 2007; Laursen et al., 2009), distinct SERCA conformational states were determined at atomic resolution.

A very potent and highly selective inhibitor is thapsigargin (TG), a sesquiterpene lactone derived from the plant *Thapsia garganica* (Rasmussen et al., 1978). TG is the most widely employed SERCA inhibitor (Michelangeli and East, 2011) and can inhibit SERCA activity with an IC_50_ in the sub-nanomolar range (Sagara and Inesi, 1991). Other specific SERCA inhibitors are cyclopiazonic acid (CPA) (Seidler et al., 1989), a secondary metabolite from certain fungi, and the synthetic compound 2,5-di(tert-butyl)hydroquinone (DBHQ) (Moore et al., 1987). Mutational analysis and crystallographic data have shown that CPA and DBHQ occupy the same binding pocket at the cytoplasmic ends of the transmembrane helices TM1–TM4, while TG binds in a groove delimited by TM3, TM5, and TM7 (for a review see Yatime et al., 2009), where the residue Phe256 plays a fundamental role for both binding and inhibitory effect of TG (Xu et al., 2004). TG, CPA, and DBHQ (Table 1) affect SERCA transport activity in a similar way. These inhibitors bind to SERCA in a calcium-free E_2_ conformation and stabilize a compact ATPase conformational state (dead-end state), preventing cytoplasmic calcium binding and catalytic activation (Inesi et al., 2005; Yatime et al., 2009; Michelangeli and East, 2011).

**Table 1:** SERCA inhibitors and activators cited in the text.

	**Compounds**	**Main properties and therapeutic applications**	**References**
**Inhibitors**	Thapsigargin (TG)	Very potent and highly selective inhibitor. TG-related prodrugs as anticancer drugs.	Sagara and Inesi, 1991; Denmeade et al., 2012; Doan et al., 2015
	Cyclopiazonic acid (CPA)	Potent and specific inhibitor. Cardioprotective effect. CPA derivatives as antimalarial agents.	Seidler et al., 1989; Avellanal et al., 1998; Moncoq et al., 2007; Cardi et al., 2010
	2,5-di(tert-butyl)hydroquinone (DBHQ)	Specific inhibitor.	Moore et al., 1987; Obara et al., 2005
	1,3-dibromo-2,4,6-tris (methyl-isothio-uronium) benzene (Br_2_-TITU)	SERCA and Na^+^,K^+^-ATPase inhibitor.	Berman and Karlish, 2003
	Cisplatin	Widely employed platinum-containing anticancer drug. SERCA and Na^+^,K^+^-ATPase inhibitor.	Wang and Lippard, 2005; Tadini-Buoninsegni et al., 2017
	Curcumin	Antioxidant, anti-inflammatory and anticancer effects. Antimalarial activity.	Bilmen et al., 2001; Reddy et al., 2005; Schaffer et al., 2015
**Activators**	Istaroxime	Cardiac drug with inotropic and lusotropic properties. Stimulatory effect on cardiac SERCA2a isoform. Therapeutic applications in acute and chronic heart failure.	Rocchetti et al., 2005; Micheletti et al., 2007; Gheorghiade et al., 2011; Ferrandi et al., 2013
	Pyridone derivative	Stimulatory effect on cardiac SERCA2a isoform. Potential therapeutic applications in heart failure.	Kaneko et al., 2017
	CDN1163	Allosteric SERCA activator. Potential pharmacological agent for diabetes and metabolic dysfunction.	Cornea et al., 2013; Gruber et al., 2014; Kang et al., 2016

The inhibitory effects of TG, CPA, DBHQ, and1,3-dibromo-2,4,6-tris (methyl-isothio-uronium) benzene (Br_2_-TITU), another SERCA inhibitor (Berman and Karlish, 2003), on Ca^2+^-ATPase transport activity were also characterized by electrophysiological measurements on a SSM (Tadini-Buoninsegni et al., 2008b). In this study it was shown that Br_2_-TITU displays an inhibitory mechanism different from that of TG, CPA, and DBHQ. In particular, it was demonstrated that the inhibitory effect of Br_2_-TITU is related to kinetic interference with a conformational transition of the phosphorylated intermediate (E_1_P-Ca_2_ to E_2_P transition).

It is noteworthy that TG-related prodrugs are being evaluated as anticancer drugs. Since TG will inhibit SERCA proteins regardless of the cell type thereby damaging intracellular calcium homeostasis not only in cancer cells but also in normal cells, its high cytotoxicity prevents direct use of TG as a general antitumor agent. However, a TG-based prodrug strategy was developed to overcome the above-mentioned limitation (Denmeade et al., 2012; Andersen et al., 2015; Doan et al., 2015; Cui et al., 2017). In particular, a prodrug, named mipsagargin, was obtained by conjugating a TG analog to a peptide that is targeted by prostate-specific membrane antigen (PSMA) (Denmeade et al., 2012; Andersen et al., 2015; Doan et al., 2015), which is overexpressed in prostate cancer cells and most tumor endothelial cells. This inactive and non-toxic prodrug becomes activated once it reaches tumor cells and the specific peptide sequence is cleaved by PSMA, thereby releasing the active cytotoxic TG analog. The prodrug mipsagargin can therefore be considered as a potential therapeutic agent for the treatment of various types of cancer, including prostate, breast and bladder cancers.

**Figure 2:**
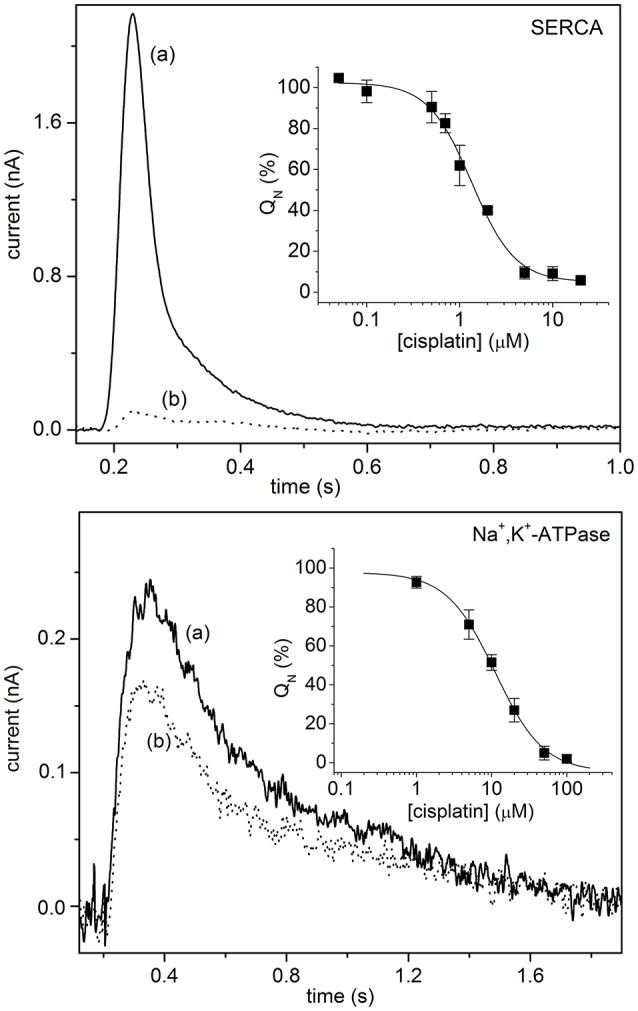
**Upper Panel:** SERCA current signals induced by 100 μM ATP concentration jumps in the presence of 10 μM free Ca^2+^ and in the absence (control measurement, solid line, a) or in the presence of 5 μM cisplatin (dotted line, b). (*Inset*) Normalized charges (Q_N_) related to ATP concentration jumps in the presence of Ca^2+^ ions as a function of cisplatin concentration. The charges are normalized with reference to the maximum charge attained in the absence of cisplatin (control measurement). The solid line represents the fitting curve to the ATP-induced charges (IC_50_ = 1.3 ± 0.1 μM). The error bars represent S.E. of three independent measurements. **Lower Panel:** Na^+^,K^+^-ATPase current signals induced by 100 μM ATP concentration jumps in the presence of 80 mM NaCl and 50 mM KCl, and in the absence (control measurement, solid line, a) or in the presence of 5 μM cisplatin (dotted line, b). (*Inset*) Normalized charges (Q_N_) related to ATP concentration jumps in the presence of Na^+^ and K^+^ ions as a function of cisplatin concentration. The charges are normalized with respect to the maximum charge measured in the absence of cisplatin (control measurement). The solid line represents the fitting curve to the ATP-induced charges (IC_50_ = 11.1 ± 0.8 μM). The error bars represent S.E. of three independent measurements. Tadini-Buoninsegni et al. (2017)—Reproduced by permission of The Royal Society of Chemistry.

In the context of antitumor agents, SSM-based electrophysiology was employed to analyze the interaction of metal-based anticancer drugs with P-type ATPases, i.e., SERCA, Na^+^,K^+^-ATPase and Cu^+^-ATPases (Sadafi et al., 2014; Tadini-Buoninsegni et al., 2014, 2017). In particular, the inhibitory effect of cisplatin (Table 1) on the transport activity of SERCA and Na^+^,K^+^-ATPase was very recently investigated (Figure 2) (Tadini-Buoninsegni et al., 2017). Cisplatin is a platinum-containing anticancer drug, which is widely employed as a chemotherapeutic agent against several tumors (Wang and Lippard, 2005). However, cisplatin administration causes inevitable adverse effects, which include nephrotoxicity, ototoxicity and neurotoxicity. We have shown that cisplatin is able to inhibit ATP-dependent cation translocation by SERCA and Na^+^,K^+^-ATPase with different degrees of potency (Figure 2). In particular, cisplatin was found to be a much stronger inhibitor of SERCA (IC_50_ of 1.3 μM) than of Na^+^,K^+^-ATPase (IC_50_ of 11.1 μM). We propose that cisplatin inhibition of SERCA and Na^+^,K^+^-ATPase activities may be relevant to the molecular mechanisms that underlie the various adverse effects of cisplatin and other platinum-containing anticancer drugs.

As concerns other specific inhibitors of SERCA activity, CPA has been proposed to have therapeutic properties. In a study of the isolated rabbit heart CPA was found to have a cardioprotective effect on myocardial ischemia (Avellanal et al., 1998). The mechanism involved in CPA cardioprotection is not fully understood and could be partly attributed to a decreased SR calcium contribution to the calcium overload induced by ischemia.

It is noteworthy that CPA has been found to be a potent inhibitor of the SERCA ortholog PfATP6 of *Plasmodium falciparum* (Cardi et al., 2010; Arnou et al., 2011), the protozoan parasite causing malaria which is responsible for most malaria-related deaths globally. Considering that the parasite *P. falciparum* is becoming increasingly resistant to some of the most commonly used antimalarial drugs, PfATP6 has been validated as a potential and promising target for the development of new and effective antimalarials. PfATP6 and mammalian SERCA are characterized by a different pharmacological profile: compared to rabbit SERCA1a isoform from the skeletal muscle, PfATP6 is less sensitive to TG and DBHQ and exhibits a much higher affinity for CPA (Cardi et al., 2010; Arnou et al., 2011). Moreover, it was shown that PfATP6 is not inhibited by the widely employed antimalarial drug artemisinin (Cardi et al., 2010; Arnou et al., 2011), which was proposed to target PfATP6 (Eckstein-Ludwig et al., 2003). In a recent study, the interaction of CPA with PfATP6 and SERCA was characterized by molecular dynamics simulations (Di Marino et al., 2015). This study points to significant differences in the mode of CPA binding to the plasmodial and mammalian SERCA. These are useful information that can assist in the design and development of CPA derivatives, which are selective toward PfATP6 and with a reduced activity against mammalian SERCA. Besides PfATP6, the *P. falciparum* protein PfATP4, a P-type Na^+^-ATPase in the plasma membrane of the parasite, has emerged as a potential antimalarial drug target and inhibition of this pump is also being considered as a possible treatment against malaria (Turner, 2016).

Among SERCA inhibitors of pharmacological interest, it is worth mentioning the polyphenolic compound curcumin (Table 1). Curcumin, which is obtained from the spice turmeric, is known for its antioxidant, anti-inflammatory and anticancer effects (Schaffer et al., 2015). It was reported that curcumin inhibits SERCA activity with an IC_50_ in the micromolar range (Bilmen et al., 2001; Dao et al., 2016) by stabilizing the E1 conformational state of SERCA and preventing ATP binding and ATP-dependent phosphoenzyme formation (Bilmen et al., 2001). In the search for new antimalarial agents, curcumin was shown to possess a remarkable antiplasmodial activity, as demonstrated by the inhibitory effects of curcumin on a chloroquine-resistant *P. falciparum* strain (Reddy et al., 2005). It was proposed that the PfATP6 protein could be a possible target for curcumin antimalarial action (Reddy et al., 2005). In a very recent study, selected curcumin analogs were synthesized and their antimalarial activity against different *P. falciparum* strains was evaluated (Dohutia et al., 2017). In particular, molecular docking was performed to investigate the interaction of these curcumin analogs with PfATP6. The results of this study may provide useful information for the development of curcumin derivatives which could serve as promising drug candidates against malaria.

## Pharmacological stimulation of SERCA activity

Molecules that are able to stimulate SERCA activity have been recently identified (Table 1). A remarkable example of SERCA activators is the drug istaroxime. Istaroxime is an innovative cardiac drug which combines inotropic (cardiomyocyte contraction) and lusotropic (cardiomyocyte relaxation) properties (Hasenfuss and Teerlink, 2011). Istaroxime has a double mechanism of action, i.e., it inhibits Na^+^,K^+^-ATPase activity and exerts a stimulatory effect on SERCA2a (Micheletti et al., 2002, 2007; Rocchetti et al., 2005, 2008), the SERCA isoform in the heart which is central to cardiac electrophysiological and mechanical function. Preclinical studies and clinical trials indicate that combining SERCA2a stimulation and Na^+^,K^+^-ATPase inhibition may increase contractility and facilitate active relaxation, improving both systolic and diastolic heart function (Gheorghiade et al., 2011). The stimulatory effect of istaroxime on SERCA activity was investigated by combining different experimental methods, including electrophysiological measurements on dog cardiac SR vesicles adsorbed to a SSM (Ferrandi et al., 2013). This study shows that istaroxime enhances SERCA2a activity, Ca^2+^ uptake, and Ca^2+^-dependent charge movements into SR vesicles from healthy or failing dog hearts. It was proposed that istaroxime acts by displacing the regulatory protein phospholamban (PLN) from the SERCA2a/PLN complex, thereby removing the inhibitory effect of PLN on this complex. Displacement of PLN from SERCA2a may favor the SERCA2a conformational transition E_2_ to E_1_, thus accelerating Ca^2+^ cycling. Istaroxime with its unique mode of action may provide a new small-molecule therapeutics for the treatment of both acute and chronic heart failure.

Very recently, a pyridone derivative was reported to activate the SERCA2a isoform by attenuating the inhibitory effect of PLN (Kaneko et al., 2017). *In vitro* and *in vivo* experiments have demonstrated that the pyridone derivative stimulates the Ca^2+^-dependent ATPase activity of cardiac SR vesicles, increases Ca^2+^ transients of isolated adult rat cardiomyocytes and accelerates contraction and relaxation of isolated perfused rat hearts. It was concluded that the pyridone derivative behaves like a SERCA2a activator, which binds and inhibits PLN, and enhances systolic and diastolic functions of the heart. The pyridone derivative is thus proposed as a novel lead compound for therapeutic applications in heart failure.

Impaired SERCA function leads to elevation of intracellular calcium concentration and alterations in calcium homeostasis, which trigger endoplasmic reticulum (ER) stress. ER stress is associated with a variety of common diseases (Oyadomari and Mori, 2004), including the metabolic syndrome and type 2 diabetes (Back and Kaufman, 2012). Pharmacological activation of SERCA can reduce ER stress and may therefore represent a novel strategy for the treatment of diabetes and metabolic disorders. A recent study (Kang et al., 2016) evaluated the metabolic effects of the quinoline-amide compound CDN1163, which is a novel SERCA activator (Cornea et al., 2013; Gruber et al., 2014). CDN1163 directly binds to the SERCA enzyme to activate Ca^2+^-ATPase activity, probably via an allosteric mechanism (Cornea et al., 2013; Gruber et al., 2014). Kang et al. (2016) demonstrated that CDN1163 activation of the SERCA2b isoform from mouse liver reduces ER stress and improves mitochondrial efficiency and metabolic parameters in an animal model of insulin resistance and type 2 diabetes (*ob/ob* mice), suggesting that SERCA activators may represent promising pharmacological agents to treat diabetes and metabolic dysfunction. Finally, since defective SERCA is a significant cause of ER stress and neuron loss in Parkinson's disease, direct activation of SERCA by small-molecule drugs is currently explored as a viable strategy to develop a novel therapeutic approach to cure Parkinson's disease (Dahl, 2017).

## Conclusions

SERCA plays an essential role in the maintenance of cellular calcium homeostasis and calcium pump malfunction is associated to severe disorders and several pathophysiological conditions. Therefore, the SERCA enzyme represents an important target for the development of new drugs. SERCA inhibitors have been identified as potential drug candidates against various diseases. As discussed above, inhibition of SERCA activity in cancer cells provides an alternative therapeutic approach to cure different types of cancer. Moreover, specific inhibitors targeting the SERCA ortholog PfATP6 in the parasite *P. falciparum* may serve as innovative and effective antimalarial agents. In addition, studies on novel SERCA activators indicate that pharmacological activation of SERCA may constitute a promising strategy for the treatment of heart failure and diabetes. Research effort is devoted to the synthesis of highly selective and potent drugs, that can target SERCA in a tissue-specific manner. As most tissue/cell types can express more than one SERCA isoform, it would be highly desirable, although very challenging, to develop isoform-specific drugs that can interact selectively with one SERCA isoform, leaving the other protein molecules intact (Yatime et al., 2009; Michelangeli and East, 2011).

## Author contributions

FT-B: Wrote the manuscript and revised it critically for important intellectual content; SS, RG, and MM: Edited the manuscript and revised it critically for important intellectual content; SS: Helped FT-B in preparing the figures. All authors have read and agreed with the final version of the manuscript.

### Conflict of interest statement

The authors declare that the research was conducted in the absence of any commercial or financial relationships that could be construed as a potential conflict of interest.

## Abbreviations

Br_2_-TITU: 1,3-dibromo-2,4, 6-tris(methyl-isothio-uronium) benzene
DBHQ: 2,5-di(tert-butyl)hydroquinone
CPA: cyclopiazonic acid
ER: endoplasmic reticulum
PLN: phospholamban
PSMA: prostate-specific membrane antigen
SR: sarcoplasmic reticulum
SERCA: sarco(endo)plasmic reticulum Ca^2+^-ATPase
SSM: solid supported membrane
TG: thapsigargin.
